# LC–MS based metabolic fingerprinting of apricot pistils after self-compatible and self-incompatible pollinations

**DOI:** 10.1007/s11103-020-01098-5

**Published:** 2020-12-09

**Authors:** József Lénárt, Attila Gere, Tim Causon, Stephan Hann, Mihály Dernovics, Olga Németh, Attila Hegedűs, Júlia Halász

**Affiliations:** 1 Department of Applied Chemistry, Faculty of Food Science, Szent István University, Villányi út 29-43, Budapest, 1118 Hungary; 2 Department of Genetics and Plant Breeding, Faculty of Horticultural Science, Szent István University, Ménesi út 44, Budapest, 1118 Hungary; 3 Department of Postharvest Sciences and Sensory Evaluation, Faculty of Food Science, Szent István University, Villányi út 29-43, 1118 Budapest, Hungary; 4 Institute of Analytical Chemistry, University of Natural Resources and Life Sciences, Muthgasse 18, 1190 Vienna, Austria; 5 Department of Plant Physiology, Agricultural Institute, Centre for Agricultural Research, Brunszvik u. 2, Martonvásár, 2462 Hungary

**Keywords:** Apricot, Mass spectrometry, Metabolic fingerprinting, Metabolomics, Self-incompatibility

## Abstract

**Key message:**

LC-MS based metabolomics approach revealed that putative metabolites other than flavonoids may significantly contribute to the sexual compatibility reactions in *Prunus armeniaca*. Possible mechanisms on related microtubule-stabilizing effects are provided.

**Abstract:**

Identification of metabolites playing crucial roles in sexual incompatibility reactions in apricot (*Prunus armeniaca* L.) was the aim of the study. Metabolic fingerprints of self-compatible and self-incompatible apricot pistils were created using liquid chromatography coupled to time-of-flight mass spectrometry followed by untargeted compound search. Multivariate statistical analysis revealed 15 significant differential compounds among the total of 4006 and 1005 aligned metabolites in positive and negative ion modes, respectively. Total explained variance of 89.55% in principal component analysis (PCA) indicated high quality of differential expression analysis. The statistical analysis showed significant differences between genotypes and pollination time as well, which demonstrated high performance of the metabolic fingerprinting and revealed the presence of metabolites with significant influence on the self-incompatibility reactions. Finally, polyketide-based macrolides similar to peloruside A and a hydroxy sphingosine derivative are suggested to be significant differential metabolites in the experiment. These results indicate a strategy of pollen tubes to protect microtubules and avoid growth arrest involved in sexual incompatibility reactions of apricot.

**Electronic supplementary material:**

The online version of this article (10.1007/s11103-020-01098-5) contains supplementary material, which is available to authorized users.

## Introduction

Genetic variation creates the basis of evolutionary success of a species allowing natural selection to favor the genotypes most adapted to external conditions. In sexually-reproducing organisms, the sources of genetic variation include mutations and several processes in meiosis. However, in hermaphrodite plants, self-fertilization may result in a serious loss of genetic variability and consequent inbreeding depression (Good-Avila et al. 2008; Martínez-García et al. 2012). Such plants have elaborated genetically controlled mechanisms to prevent self-fertilization (Kao and Tsukamoto 2004). One such mechanism is the gametophytic self-incompatibility (GSI) in the Rosaceae family determined by the self-incompatibility (*S*) locus. Two genes were identified in the *S*-locus with one coding a pistil-specific ribonuclease enzyme (*S*-RNase) and another for an *S*-haplotype-specific F-box (SFB) protein specifically produced in pollen tubes (Matsumoto and Tao 2019). Self/non-self-recognition in these plants is mediated by these molecules. Non-self *S*-RNases enter the compatible pollen tubes where they are rapidly degraded through a general inactivation mechanism and hence protein biosynthesis is undisturbed. In contrast, self *S*-RNases are recognized and prevented from degradation (Matsumoto and Tao 2016; Sonneveld et al. 2005), which results in an incompatible reaction without fruit set.

The precise molecular interactions behind the (in)compatibility reaction and between the *S*-RNase and SFB are unknown. Interestingly, solanaceous plants also exhibit an *S*-RNase-based GSI system and vacuole stability was reported as a crucial element in the physiological reactions leading to compatible or incompatible mating (Goldraij et al. 2006; McClure 2006). In solanaceous plants, besides *S*-RNase and SFB, additional non-*S*-specific proteins (e.g., HT-B, 120 K, NaTTS) were determined to play a pivotal role in the incompatibility response. Mutant phenotypes or transgenic experiments allowed clarification that loss-of-function of such factors results in the breakdown of the self-incompatibility barrier in a non-*S*-specific manner. Although such proteins have not been identified in *Prunus* until now, non-*S*-locus factors may also contribute to the incompatibility response. Cachi and Wünsch have found that the locus associated with self-compatibility in ‘Cristobalina’ sweet cherry is located in LG3, in contrast with the *S*-locus in LG6 (Cachi and Wünsch 2011). A loss-of-function mutation in a non-*S* locus gene, also mapped on LG3, was shown to induce self-compatibility in the apricot cultivars ‘Canino’ and ‘Katy’(Muñoz-Sanz et al. 2017; Zuriaga et al. 2012, 2013).

Self-incompatibility and mutations rendering self-compatibility of specific cultivars have been long known in apricot (*Prunus armeniaca* L.) (Halász et al. 2014, 2007; Muñoz-Sanz et al. 2017). Proteomic differences were also reported after self- and cross-pollinated self-incompatible (SI) apricots by two-dimensional polyacrylamide gel electrophoresis (2D-PAGE) and liquid chromatography-electrospray tandem mass spectrometry (LC–ESI–MS/MS) (Feng et al. 2006). In a subsequent study, the same authors extended the number of proteins identified in apricot pistil after self- and cross-pollination of SI genotypes (Feng et al. 2009). A recent study performed transcriptome analysis in almond pistils after compatible and incompatible pollinations and identified 1357 differentially expressed genes involved in metabolic processes and binding molecular functions, which may have a role in GSI (Gómez et al. 2019).

Metabolomics provides a useful practical tool for bridging the genotype–phenotype gap in plants (Hall and Hardy 2012). The metabolome represents all of the relevant primary and secondary metabolites, typically with molecular masses < 1.5–2 kDa, in a given organism. The composition of the metabolome is regarded as a unique fingerprint, which dynamically changes in response to external effects. Consequently, metabolic fingerprinting can give a closer hint how the final phenotypes evolve. Liquid chromatography combined with mass spectrometry (LC–MS) based metabolic fingerprinting is proven to be a powerful tool to identify significant differentiating compounds and related metabolic changes in plants (Atkinson et al. 2012; Rodrigues-Neto et al. 2018). There are some databases which are specific to plant metabolites and serve as good sources for substance identification in plant metabolomics, e.g., Dictionary of Natural Products (Quinn et al. 2008) contains biologically active small molecules and MoTo DB database (Moco et al. 2006), which is specific to tomato metabolites.

Still, metabolite identification is the bottleneck of LC–MS based metabolic fingerprinting. The number of metabolites with accurate mass LC–MS and LC–MS/MS data is relatively limited in metabolite databases compared to libraries intended for GC–MS based identification (Weckwerth et al. 2004; Xiao et al. 2013), especially when in silico prediction of LC–MS/MS fragmentation fails to provide unambiguous confirmation. Moreover, pollination/germination related issues are seldom covered in metabolomics, as pistils are hardly targeted with LC–MS based metabolomic tools because of the highly time-dependent characteristics (Qin et al. 2011); such analyses are mostly limited to flavonoid research (Chen et al. 2012; Hanhineva et al. 2008).

Indeed, there have been only a few studies dealing with non-flavonoid targeted research in pollination metabolomics in the field of Rosaceae. Cubells-Baeza and co-workers attempted to identify the ligand of Pru p 3, a peach lipid transfer protein (LTP) (Cubells-Baeza et al. 2017). However, the study could directly focus on one molecule that could be highly purified through orthogonal chromatographic steps, the instrumental set-up of a MALDI coupled to an ESI-QTOF HR (high resolution) mass spectrometer could only prove that the ligand is a derivative of camptothecin. Facing the need for milligrams of the target analyte to enable NMR-based identification, such a conclusion without reporting an ultimate structure can be regarded as a common event. Concerning a higher level of metabolomics, that is, unknown screening for a non-limited set of metabolites, there has been only one study on Rosaceae: ^1^H-NMR-profiling results of non-pollinated and pollinated styles of pear (*Pyrus communis* L.) have been recently reported (Mandrone et al. 2019). The authors could finally frame a set of four molecular classes (e.g., molecules with a kaempferol moiety) and 15 basic (known) molecules that showed relation with pollination metabolism. Clearly, ^1^H-NMR-profiling can definitely function as a preliminary screening to shorten the list of potential candidates for a more targeted study, but it is unable to assess the complexity of metabolites and to pinpoint single key molecules—if there are any.

To the best of our knowledge, there have been no prior attempts to deduce the self-incompatibility reactions in Rosaceae fruit tree species by LC–MS based metabolic fingerprinting. Therefore, the aim of the present study was to compare the metabolic fingerprints of self-pollinated pistils of a SC and SI apricot cultivar to shed light on physiological differences between incompatible and compatible reactions in the *Prunus S*-RNase-based GSI system. We investigated whether biomarkers or key metabolites participating in these reactions can be found and assigned by LC–MS based metabolic fingerprinting, which may explain currently unknown aspects of the (in)compatibility reaction.

## Materials and methods

### Chemicals and reagents

Tebuconazole (applied as internal standard) and formic acid were purchased from the Merck-Sigma group (St. Louis, MO, USA). HPLC gradient grade acetonitrile, methanol, and HPLC grade ethanol were ordered from Fisher Scientific (VWR, Radnor, PA, USA). Ultra-pure water (> 18 MΩ• cm) was obtained from a Merck-Millipore Milli-Q system (Bedford, USA). Peloruside A authentic standard material was gained by chemical synthesis and also by isolation from *Mycale hentscheli* marine sponge samples with the kind contribution of Dan Sackett (Eunice Kennedy Shriver National Institute of Child Health and Human Development, Bethesda, USA) and Peter Northcote (Victoria University of Wellington, Wellington, New Zealand) and their workgroups, respectively. Peloruside A stocks were prepared in ethanol and were stored at − 18 °C until analysis.

### Plant material

Two apricot (*Prunus armeniaca* L.) genotypes were studied, ‘Ceglédi óriás’ (*S*_8_*S*_9_) and ‘Pannónia’ (*S*_C_*S*_C_) as SI and SC species, respectively (Halász et al. 2007). Branches (with flower buds) were collected from apricot trees in the orchard (Szigetcsép, Hungary; 47°15′30″N 18°58′10″E) and were subsequently transported into the laboratory and kept in tap water. Collection was performed when flowers were in the balloon stage (pollen grains were not released from the anthers and hence spontaneous pollination was not possible). In a subsequent step, the flowers were emasculated and controlled hand-pollination was performed by collecting pollen grains from the desiccated anthers. After pollination, pistils were collected after 3, 24 and 96 h. Pistils of both species without pollination were also collected and used as a control (0 h). Samples from different species and pollination stages were collected and immediately frozen in liquid nitrogen and stored at − 80 °C until analysis.

### Sample preparation

The pistils from the fridge were immediately frozen again in liquid nitrogen in a mortar and were disrupted by a pestle. 100 ± 1 mg portions of the gained powder were weighed on an analytical scale in sterile 1.5 mL Eppendorf tubes. Three biological replicates were made for both species in every pollination stage and a total of 24 samples were obtained. Extraction was performed in the same tubes by 1000 µL 80:20% v/v methanol:ultra-pure water, vortexed thoroughly and shaken in a thermomixer at 25 °C for 15 min. Before vortexing, 10 ng mL^−1^ tebuconazole was added as internal standard. The insoluble plant material was separated by centrifugation at 10,000×*g* at 4 °C. Ten-fold dilutions with ultra-pure water were performed on the collected supernatants and the samples were filtered through disposable 0.45 μm PTFE syringe filters before injection into the LC–MS system.

### HPLC-ESI-QTOF-MS analysis

The LC–MS coupling was carried out with an Agilent 1100 HPLC system (Agilent Technologies, Waldbronn, Germany) and a quadrupole time-of-flight (QTOF) high resolution mass spectrometer (Agilent 6530 Accurate Mass QTOF LC/MS, Palo Alto, CA, USA). 10 µL of the sample extracts were injected on a Zorbax Eclipse XDB-C18 reversed phase column (3.5 μm, 2.1 mm × 50 mm; Agilent). Water with 0.1% v/v formic acid was used as eluent A, and acetonitrile with 0.1% v/v formic acid was used as eluent B; HPLC flow was set to 400 µL min^−1^. Gradient elution was set as follows: 0–3 min 10% B; 3–15 min up to 100% B; 15–17 min 100% B, 17–18 min down to 10% B; 18–22 min 100% B. The QTOF-MS instrument was operated with an Agilent 6220 instrument-derived dual electrospray ionization (ESI) ion source in positive and negative ionization modes. Mass calibration was performed by introducing a mass calibration solution by direct infusion according to the manufacturer’s instructions; due to the continuous mass correction, the experienced mass accuracy was ~ 3 ppm and mass resolution was above 10,000 (FWHM) at *m/z* 400. The instrumental parameters and settings are summarized in the Electronic supplementary material in Table S1. All samples were first run to obtain full scan MS spectra, and in addition, some samples were run to obtain MS^2^ fragmentations using collision induced dissociation (CID) from the compounds of interest. Data were recorded both in centroid and profile modes in the range *m/z* 100–1700.

### Data mining strategy

Mass spectrometric data were recorded with Agilent Mass Hunter Data Acquisition B.04.00 and processed with Agilent Mass Hunter Qualitative Analysis software B.06.00. Untargeted compound search (general unknown screening) was performed by the Molecular Feature Extraction (MFE) algorithm. Ions are grouped together to create a given molecular feature whose extracted ion chromatograms (EICs) share similar elution profile and additionally their mass differences can be explained via isotope ratios, adduct or dimer formations or neutral losses and in-source fragmentation events. The software takes into consideration user defined parameters regarding mass accuracy, mass resolution, minimal signal and compound intensities, minimum number of ions per compound, etc.

According to the manufacturer‘s instructions the so-called recursive feature extraction protocol was applied to enhance the performance of the MFE algorithm. Minimum intensity of precursor ions was set to 5 × 10^3^ counts. MFE settings were optimized and the isotope spacing tolerance parameter was reduced from the default value (5 mDa + 7 ppm) to 2 mDa + 3 ppm. General unknown screening algorithms may result in false positive and negative hits or wrong assignment of monoisotopic masses even after optimization, consequently all metabolites of interest were verified manually regarding the isotopic pattern, adducts, in-source fragments, etc.

### Differential expression analysis

Statistical analysis of samples after untargeted compound search was done by Mass Profiler Professional (Agilent, version 12.1) and an LC–MS manufacturer independent software XLSTAT (version 2013.1; Addinsoft, USA) running as an add-in of Microsoft Excel software. Recursive feature extraction and compound alignment (including abundance normalization to internal standard) were performed by Mass Profiler Professional while further statistical analyses were carried out with both of the software packages. After compound alignment, the data were exported into comma separated (.csv) format to be handled by Microsoft Excel.

**Fig. 1:**
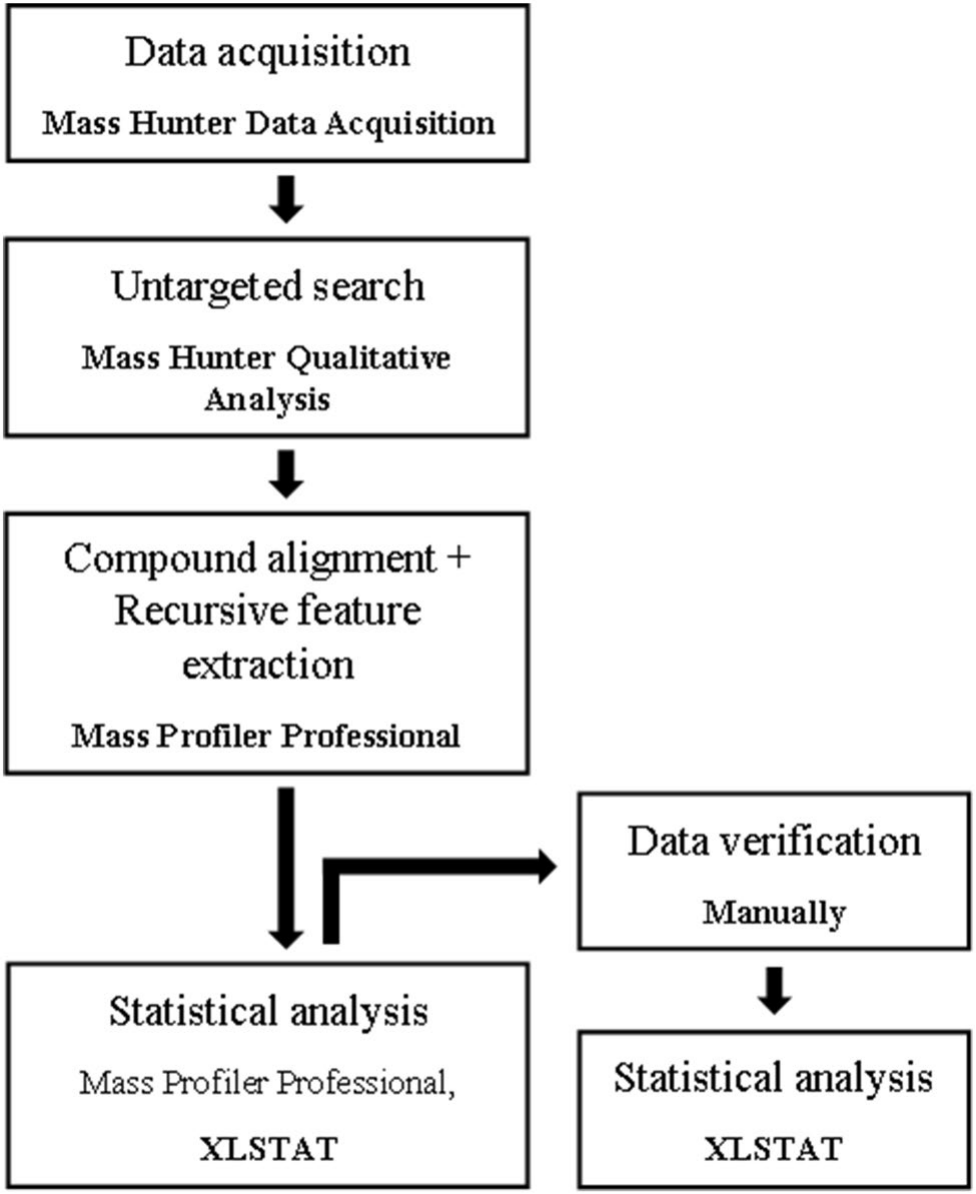
Workflow of the applied statistical differential expression analysis

The obtained differential feature list was manually verified to eliminate redundant information and to correct peak integration failures so compounds of very low intensity could be also filtered out. To be able to combine positive and negative ionization data, the final analysis was performed by XLSTAT. Workflow of the complete data evaluation can be seen on Fig. 1. Both statistical differential expression analyses consisted of the following methods: (i) filtering by frequency (features must be present in 100% of the replicates); (ii) retaining entities that monotonously increase or decrease in time; (iii) significant metabolites were determined by *t*-test at *p* < 0.001; (iv) finally a heatmap containing the differential compounds was created and principal component analysis (PCA) was performed in order to visualize how the groups are differentiated and to determine the most significant metabolites.

### Assignment of differential compounds

The elemental composition (molecular formula) of the differential metabolites was determined based on their monoisotopic mass and isotopologue ratios. Free online and commercially available compound databases and in silico fragmentation tools (including Dictionary of Natural Products (DNP); SciFinder, ChemSpider, KnapSack, MassBank, Metlin, LIPID MAPS, LipidBlast, GNPS, MetFrag and PubChem) were searched individually and with the help of the MassHunter Lipid Annotator software tool (version 1.0, build 1.0.54.0; Agilent; see the Electronic supplementary material Table S1) against the determined molecular formulae and MS/MS fragments.

The compounds of interest included in the differential feature list were analyzed in a subsequent run on the HPLC-ESI-QTOF-MS system in Auto MS/MS mode to perform fragmentation reactions by CID in order to gain structural information. The user defined parameters of the Auto MS/MS mode are summarized in Table S2 in the Electronic supplementary material.

## Results

### Detected metabolites

The applied methanolic extraction resulted in a large number of metabolites: the total numbers of aligned compounds were 4006 and 1005 in positive and negative ionization modes, respectively. Because of the large number of metabolites, manual checking of the whole list is not viable. Therefore, incorporation of the recursive feature extraction was required to achieve sufficient metabolite searching performance. Quality control in terms of repeatability by PCA was also checked and no significant outlier samples were found among parallels.

### Determination of differential metabolites: combination of software based and manual data reduction steps

Statistical analysis was performed by both software packages in order to check if there was a difference between the performance of the software. Reliability of the statistical evaluation process was indicated by the two different lists containing exactly the same entities. The numbers of differential entities were 72 and 61 in positive and negative ion modes, respectively. After merging the compounds found in both ionization modes, manual verification was performed on this list and redundant information was removed by checking the mass spectra manually at the entities` corresponding retention time (t_R_) values. When necessary, the integration failures were also corrected by manually integrating the EICs at the monoisotopic masses or, where needed, at the characteristic/abundant adducts. Metabolites having low signal-to-noise ratio and very low abundances were filtered out since their identification was not considered due to decreased mass accuracy, inaccurate isotope ratio and poor MS^2^ product ion spectra. It is also important to highlight that no dedicated list of commonly targeted compounds (e.g., highly abundant flavonoids) was set in order not to bias the statistical evaluation.

**Fig. 2:**
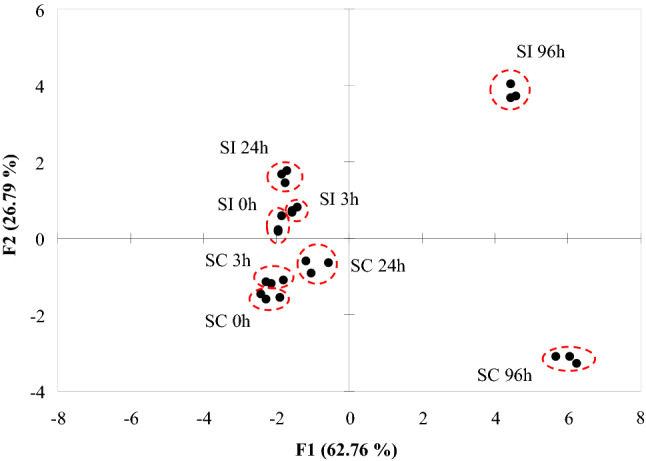
PCA illustration of components differing between incompatible and compatible pollinations and among time intervals (0, 3, 24 and 96 h) after self-pollination. Sampling time (F1) explains most of the total variance

**Fig. 3:**
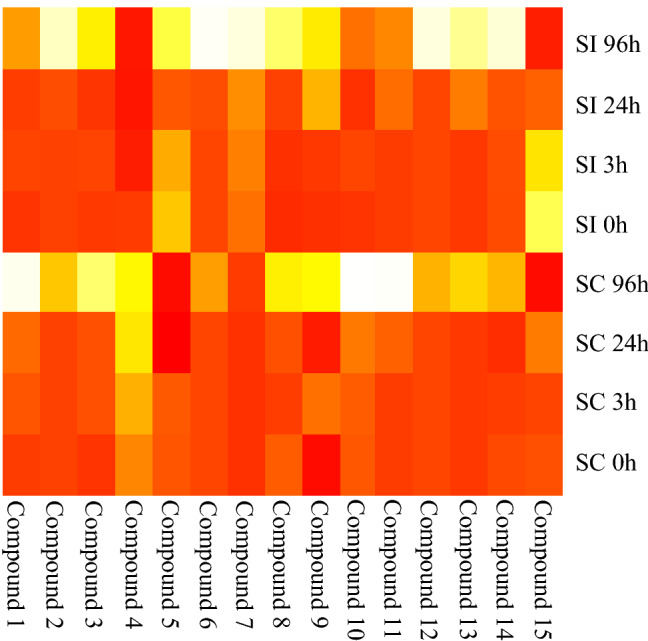
Heatmap illustration of components differing between incompatible and compatible pollinations and among time intervals (0, 3, 24 and 96 h) after self-pollination. The higher is the intensity of the compound, the darker is its colour

Focusing on the entities that showed monotonous decreasing or increasing tendency, the highest abundances of the individual metabolites were checked in the samples collected without pollination (SC 0 h and SI 0 h) or 96 h after pollination (SC 96 h and SI 96 h), respectively. Finally, 15 metabolites were found to be significant. Both the PCA and heatmap indicated reliable differentiation of the samples either by (in)compatible pollination or sampling time after pollination (Figs. 2, 3).

For the PCA, no computed variables were used, all variables measured the construct in the same direction, appropriate correlations were present in the dataset and the Kaiser–Meyer–Olkin measure of sampling adequacy proved to be 0.738, which is interpreted as good. The first two factors explain 89.55% of the total variance (F1:62.76%, F2:26.79%).

Main contributors to F1 and F2 are presented in Table 1. The cultivars are differentiated along F2 but the sampling time is differentiated along F1. Based on this observation, we concluded that the metabolites having the highest contribution to F2 could be the most responsible for sexual incompatibility reactions. These metabolites were compounds #4, #5 and #7 having neutral masses of 548.3198, 204.0871 and 315.2773 Da at t_R_ 10.07, 0.45 and 9.53 min, respectively.

**Table 1:** Squared cosine values of the individual compounds to the principal components in PCA

Compound number	Contribution to F1	Contribution to F2
# 1	**0.595**	0.346
# 2	**0.935**	0.045
# 3	**0.876**	0.063
# 4	0.002	**0.803**
# 5	0.061	**0.785**
# 6	**0.833**	0.131
# 7	0.373	**0.594**
# 8	**0.957**	0.001
# 9	**0.734**	0.003
# 10	0.400	**0.542**
# 11	**0.518**	0.363
# 12	**0.886**	0.081
# 13	**0.947**	0.025
# 14	**0.881**	0.099
# 15	**0.417**	0.138

Bold values indicate the highest cosine values of the actual compound

### Metabolite identification strategy

**Fig. 4:**
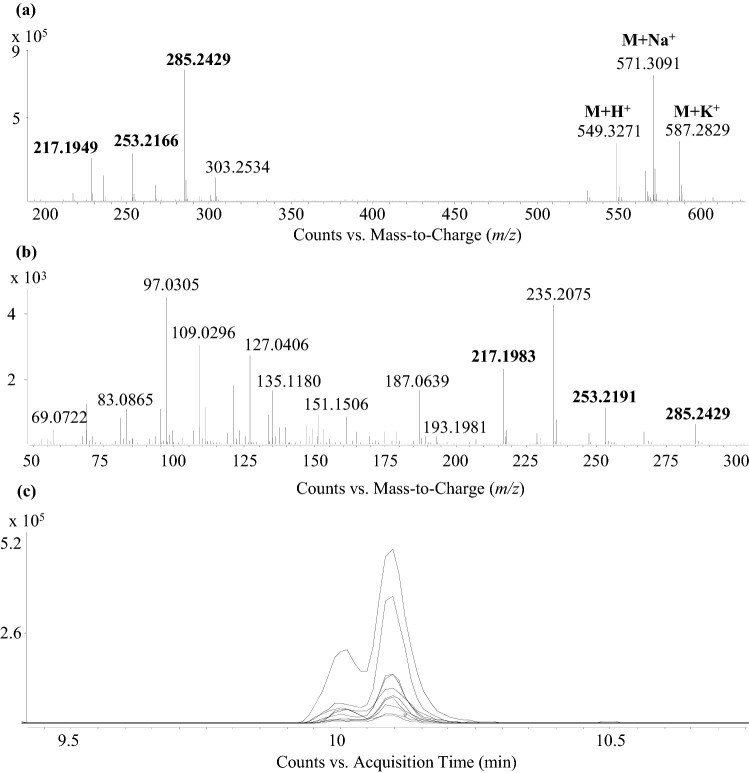
Occurrence of the common mass signals in the full scan spectrum (**a**) and MS^2^ spectrum at t_R_ 10.07 min of *m/z* 549.3271 (**b**) proves the presence of in-source fragments (**c**). Mass values highlighted in bold indicate common fragments. This observation enabled to determine m/z 549.3271 to be the [M + H]^+^ form of compound #4

Showing the highest contribution, compound #4 was found to be the most differentiating compound and the most intensive one in the SI species in positive ion mode (also found in negative ion mode as a differentiating metabolite). During the examination of MS spectra at t_R_ 10.07 min (Fig. 4a), several in-source fragments (m/z 217.1949, 253.2166, 285.2429) and adducts ([M + Na]^+^ at *m/z* 571.3091 and [M + K]^+^ at *m/z* 587.2829) of compound #4 were observed. Perfect similarity of the EICs of in-source fragments indicated their shared origin which was further enhanced by their occurrence in MS^2^ spectra of the precursor *m/z* 549.3271 (Fig. 4b, c). Based on these observations, *m/z* 549.3269 (average value) was proved to be the protonated form of compound #4. As a result of molecular formula generation (MFG), 18 molecular formulae were found to be potential candidates (nitrogen rule and RDBE were considered, see Table 2).

**Table 2:** Possible molecular formulae of compound #4

Molecular formula [M + H]^+^	Theoretical *m/z* value	Δ ppm	MFG score	RDBE value^a^
C_27_H_49_O_11_	549.3269	0.7	99.14	4
C_22_H_46_N_8_O_6_P	549.3272	0.9	98.22	5
C_21_H_50_N_4_O_10_P	549.3259	1.3	96.81	0
C_24_H_41_N_10_O_5_	549.3256	2.1	95.88	10
C_24_H_53_N_6_P_4_	549.3277	1.9	95.54	4
C_25_H_37_N_14_O	549.3269	0.2	95.35	15
C_24_H_51_N_6_O_2_P_2_S	549.3264	1.1	94.99	4
C_31_H_51_O_4_P_2_	549.3257	1.4	93.66	8
C_19_H_38_N_18_P	549.3259	2.0	93.36	11
C_17_H_43_N_16_OP_2_	549.3275	1.0	93.20	6
C_28_H_45_N_4_O_7_	549.3283	3.1	92.43	9
C_25_H_52_N_4_O_3_P_3_	549.3247	3.5	92.05	4
C_23_H_42_N_12_O_2_P	549.3286	3.3	91.97	10
C_30_H_50_N_2_O_3_PS	549.3274	1.1	91.89	8
C_20_H_49_N_6_O_9_S	549.3276	1.2	91.68	0
C_32_H_46_N_4_P_2_	549.3271	0.9	90.60	13
C_17_H_41_N_16_O_3_S	549.3263	1.8	89.80	6
C_23_H_45_NO_9_	549.3243	4.4	89.79	5

^a^RDBE values refer to neutral molecular formulae

In order to filter out the correct one from the list, the molecular formula of the in-source fragment (*m/z* 285.2425) was considered, which was found to be C_17_H_33_O_3_ (theoretical *m/z* value: 285.2424, Δ = 0.2 ppm, MFG score = 99.45). Other candidates for this in-source fragment had far worse parameters. Taking this information into account, molecular formulae for compound #4 having less than 17 carbon atoms were excluded. In a subsequent step the approximate number of carbon atoms were determined based on the (A + 1)/A ratio (28.6%) and a value of 26.48 was gained (28.60/1.08). This showed that the right molecular formula must contain 25–28 carbon atoms.

From the fragments, three neutral losses of H_2_O and one of –CH_2_ could be clearly observed indicating the presence of three –OH and a methyl or –*O*-methyl groups on the C_17_ aliphatic chain. This observation provided information about the presence of at least four oxygen atoms (Table 3), consequently the hits having less oxygen could also be excluded. Finally, from the two remaining formulae C_28_H_45_N_4_O_7_ was also filtered out based on the substantially worse values of all listed parameters compared to those of C_27_H_49_O_11_ ([M + H]^+^).

**Table 3:** In-source fragments of compound #4

Fragment number	*m/z* value	Molecular formula, (M + H)^+^	Description
1	531.31591	C_27_H_47_O_10_	(M + H)^+^–H_2_O
2	285.2429	C_17_H_33_O_3_	–
3	267.2320	C_17_H_31_O_2_	–H_2_O
4	253.2166	C_16_H_29_O_2_	–
5	235.2075	C_16_H_27_O	–H_2_O
6	217.1949	C_16_H_25_	–H_2_O

Three hits based on this latter formula were found in the DNP, 27 in SciFinder and four in the ChemSpider databases, respectively. Based on the previously mentioned observations, a compound named peloruside A was supposed to be the most probable candidate, showing ten isomers from the list of 27 hits in SciFinder database. Additional information was also gained that further enhanced the probability of compound #4 being peloruside A. Namely, when examining another significant and abundant differentiating metabolite (compound #1) its molecular formula was found to be C_26_H_46_O_11_, which could correspond to peloruside B in DNP, a natural congener of peloruside A, differing in one methyl group (see Electronic supplementary material Fig. S2). These entities were found to be highly up-regulated in case of SI pollination, showing an increasing intensity over time.

**Fig. 5:**
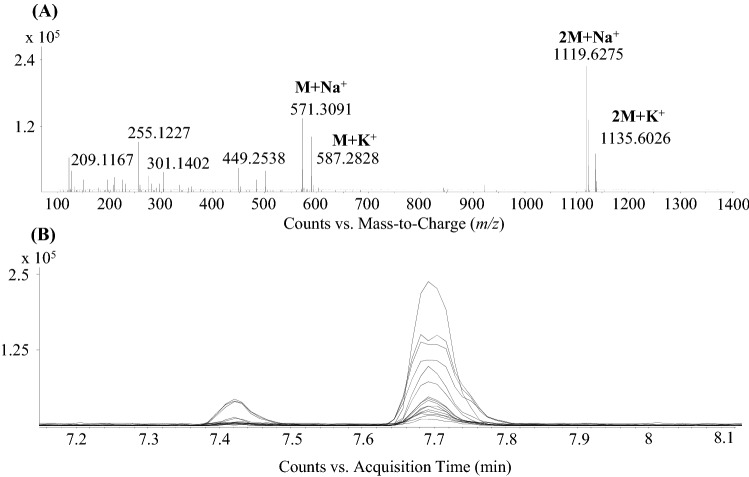
Full scan spectrum at t_R_ 7.70 min corresponding to peloruside A (**a**). In the lack of its protonated form, the sodium, potassium adducts and its dimer are the most abundant. EICs confirm that the coeluting molecules are indeed the in-source fragments of peloruside A (**b**)

Standard solutions of peloruside A (both natural and synthetic) were injected and two isomers of peloruside A were observed at 7.43 and 7.70 min both showing signals at *m/z* 571.3091, *m/z* 587.2828, *m/z* 1119.6275 and *m/z* 1135.6026 corresponding to its M + Na^+^, M + K^+^, 2 M + Na^+^ and 2 M + K^+^ forms, respectively (Fig. 5).

**Fig. 6:**
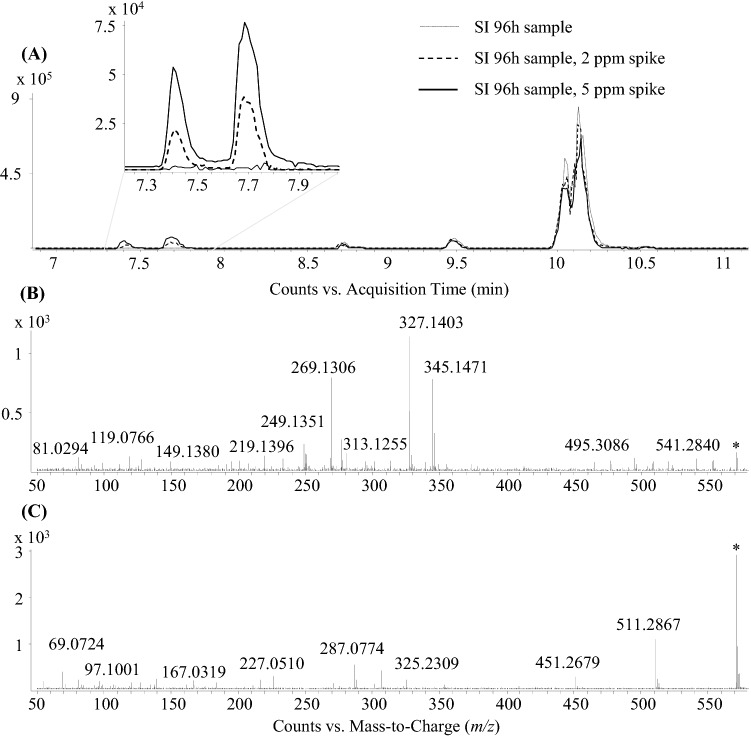
EICs of *m/z* 571.3091 in the spiked and non-spiked samples (**a**) show increasing signal intensity at t_R_ 7.43 and 7.70 min but not at the expected t_R_ 10.07 min. Differences in the MS^2^ spectra of peloruside A (**b**) and compound #4 (**c**) of their Na adducts at *m/z* 571.3091 indicate the mismatch of the two molecules

However, in a subsequent step when the SI 96 h samples were spiked with solutions containing 2 and 5 µg/mL peloruside A, increases in signal intensities in the spiked samples were also observed at 7.43 and 7.70 min, which confirmed that compound #4 was not peloruside A. MS^2^ experiments were performed where the sodium adduct was subjected to CID fragmentation (in the absence of the protonated form). MS^2^ spectra and chromatograms can be found in Fig. 6.

Based on the observations made, it can be assumed that compound #4 is not peloruside A, although based on its accurate mass, molecular formula, the dominant sodium adduct and its known microtubule stabilizing effect, it seemed to be a highly likely candidate. However, considering all of these observations, it could be putatively assumed that both compounds #1 and #4 belong also to the compound class of polyketide-based macrolides, which may have similar effects to peloruside congeners. Additional full scan and MS^2^ spectra of peloruside A and compounds #1 and #4 can be found in the Electronic supplementary material (Fig. S1–S4).

Having the second highest squared cosines value in F2, compound #5 should be also examined, but as it eluted in the void volume of the chromatographic system and showed low abundance, no attempts for identification were made.

**Fig. 7:**
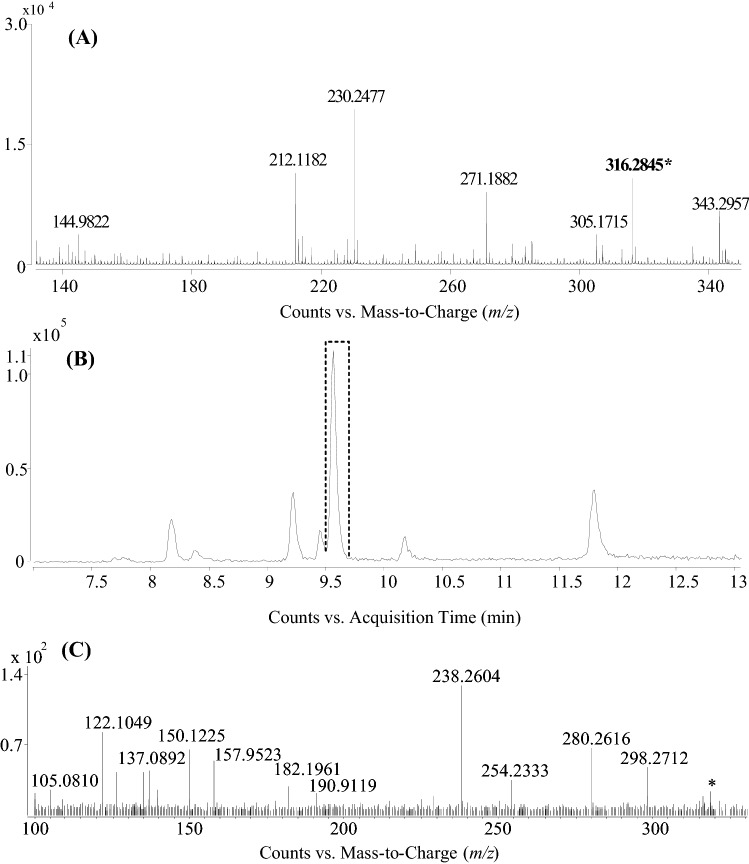
LC-ESI-QTOF-MS full scan spectrum of the *m/z* 316.2845 compound from the SC96 sample, recorded at t_R_ 9.53 min (**a**), with its extracted ion chromatogram (**b**) and MS^2^ spectrum (**c**)

The third highest squared cosines value of F2 is owned by compound #7 (Fig. 7). The molecular formula was found to be C_18_H_37_NO_3_ (theoretical M + H^+^ *m/z* 316.28462, Δppm = -0.38, t_R_ 9.53 min) and was searched against databases, taking into account the high degree of CH saturation and that negative mode data were not abundant for this compound. LIPID MAPS provided three possible hits within the class of sphingoid bases (that is, non-phosphorylated plant sphingolipids: 4-hydroxy-8cis-sphingenine, dehydrophytosphingosine and 6-hydroxysphingosine), which is supported by the fact that long-chain base type sphingolipids are ionized well in ESI positive mode (Colsch et al. 2004). MetFrag (using structures from PubChem) revealed hits with very similar structures related to basic sphingoid species, dehydrophytosphingosine and 6-hydroxysphingosine. Among these latter two compounds, none of the available experimental fragmentation patterns match that of compound #7, according to Tsugawa et al. (2019) and Yadav et al. (2003), respectively.

Altogether, compound #7 might be a member of the hydroxy sphingosine family due to the determined molecular formula, relatively high retention time, ESI+ ionization behavior and correspondence with similar database hits. Although, as no standard is commercially available for 4-hydroxy-8cis-sphingenine, it is not possible to confirm the identity of this compound without new experimental MS/MS results for the sum formula apart from what is available for dehydrophytosphingosine and 6-hydroxysphingosine.

Other differentiating compounds were not subjected to further MS^2^ experiments mostly because of their low abundance that may cause inaccuracy of *m*/*z* values and the isotopic patterns leading to ambiguous molecular formulae. It is noted that Lipid Annotator software could not support or invalidate the proposed putative identification of the compounds #1, #4 and #7 as the MS/MS fragmentation results were not corresponding to any lipid species contained in the extensive LipidBlast database also used by this software package.

## Discussion

The self-incompatibility reaction in pollen tubes of *Prunus* fruit trees is mediated by the specific recognition between pollen SFBs and pistil *S*-RNases that enter the pollen tube (Matsumoto and Tao 2019; Sonneveld et al. 2005). In incompatible pollen tubes, RNA degradation may result in the cessation of protein biosynthesis, but far less is known about the downstream physiological reactions leading to the growth arrest of incompatible pollen tubes. Compound identification is a major drawback of LC–MS based metabolic fingerprinting experiments, because the number of available metabolites in the recent databases is still limited. Although the unambiguous identification of compounds #1 and #4 was not possible due to the limited availability of specific standard compounds, our study revealed that peloruside-like compounds predominantly accumulate in self-pollinated pistils of the SI apricot cultivar ‘Ceglédi óriás’ (*S*_8_*S*_9_) and their accumulation increases throughout the first 96 h of pollen tube growth. In addition, such compounds were not detected (or had significantly lower abundance) in ‘Ceglédi óriás’ pistils without pollination.

Peloruside A, a secondary metabolite of a New Zealand marine sponge is known for having microtubule-stabilizing effects through binding to the tubulin dimers (Miller et al. 2010). In the *Papaver*-type GSI system, the massive depolymerisation of actin microfilaments upon SI stimulus is evident, which then rapidly triggers depolymerisation of microtubules (Poulter et al. 2008). Interestingly, proteomic studies revealed that several actins were detected to accumulate in apricot pistils only after incompatible pollination (Feng et al. 2009). In addition, binding of the reduced *S*-RNase to actins in *P. avium* pollen tubes was shown by yeast two-hybrid analysis and hence *S*-RNases may disrupt actin dynamics (Matsumoto and Tao 2012). Apple *S*‐RNases, by interacting with an actin-binding protein, were also found to disrupt normal F-actin circulation and reduce the growth of self-pollen tubes (Yang et al. 2018). If a link between microfilament and microtubule degradation is presumed in *Prunus* similarly to the *Papaver* GSI response, a strategy to protect microtubules with specific compounds like pelorusides seems indispensable for maintaining pollen tube activity since it was also shown that disintegrated microtubules do not reorganise later (Poulter et al. 2008).

However, another equally plausible mechanism for peloruside-like compounds in self-incompatibility might be a microtubule-targeted growth arrest. The accumulation of an agent with putative microtubule-stabilizing effects may induce cell cycle arrest, apoptosis (Hood et al. 2002), accelerated senescence (Chan et al. 2017), or the suppression of microtubule dynamics (Chan et al. 2011; Huzil et al. 2008). In addition, the uneven distribution of organelles in pollen tubes that is required for tip growth is likely a consequence of mechanisms including the regulation of myosin activity, the local fine‐scale organization of actin, and the dynamics of microtubules (Cai et al. 2015).

Regardless of the fact whether peloruside congeners are accumulating in response to a stress effect induced by self-incompatibility and help maintain the intact cytoskeleton that is essential for pollen germination and tip growth (Bove et al. 2008; Taylor and Hepler 1997) or they form a part of the degradatory mechanisms leading to pollen tube growth arrest, their exclusive occurrence in self-incompatible pollen tubes might be of crucial importance. Since it was previously determined that the pollen tube growth of incompatible apricot pollen tubes is arrested in the lower quarter of the pistils to prevent fertilization (Kodad et al. 2013), this phenomenon might be coupled with the microtubule stabilizing effects of peloruside A and its congeners. Several peloruside congeners and analogues were shown to have similar bioactivity to peloruside A (Miller et al. 2010) and hence compounds #1 and #4 that are accumulating in incompatible pollen tubes may be associated with similar physiological consequences. The metabolome analysis of pistils after incompatible and compatible pollination may shed light on the less known aspects of pollen tube growth and downstream reactions of the self/non-self-recognition in *Prunus* GSI system.

Concerning compound #7, it had been present in the pistils of both SC and SI cultivars before pollination. However, their quantities changed in a strikingly different way after self-compatible and self-incompatible pollinations. Compared to its practically unchanged levels in self-pollinated pistils of the SC cultivar ‘Pannónia’, the quantity of #7 followed a gradually decreasing trend in the pistils of self-pollinated SI plants (‘Ceglédi óriás’). This compound was proposed to be a plant sphingolipid, a member of the hydroxy sphingosine family. Sphingosine compounds were shown to take part in signalling pathways (Luttgeharm et al. 2016) and the presence of such compounds was also confirmed in *Prunus*, i.e., almond kernels (Rubino et al. 2020).

The biosynthesis of plant sphingosine compounds includes the transportation of ceramide to Golgi apparatus, where ceramidases hydrolyse them to yield sphingosine and fatty acid. In *Arabidopsis*, the *TurgOr regulation Defect 1* (*TOD1*) gene was shown to encode a Golgi-localized protein with ceramidase activity, which was preferentially expressed in pollen tubes. In addition, loss-of-function mutation of *TOD1* resulted in pollen tubes having higher than normal turgor and showing growth retardation (Chen et al. 2015). Since pollen tube growth of mutant plants could be restored by the application of sphingosine and sphingosine-1-phosphate, it confirms that sphingolipids in pollen tubes are crucial for the maintenance of turgor pressure and tip growth. *S*-RNase cytotoxicity by stopping protein biosynthesis may have a very similar physiological consequence as the loss of *TOD1* gene function, which is expected to contribute to the growth arrest of incompatible pollen tubes. However, the considerably lower quantity of such compounds in samples containing both pollen tubes and pistil tissues requires further investigation. The physiological basis of growth arrest in incompatible pollen tubes is well known (McClure et al. 1990), but a detailed mechanism leading to this phenotype is still to be elucidated. A great number (1357) of genes were shown to be differentially expressed after self-compatible and incompatible pollination in almond (Gómez et al. 2019), including the increased expression of an ATP-binding cassette (ABC) transporter G family protein coding gene in incompatible pollination. ABCG transporters are presumed to regulate lipid constituents in pistil tissues (Chang et al. 2016) and hence changes in lipids may be equally important elements or consequences of incompatibility reactions.

Further experiments including compound purification followed by NMR spectroscopy based identification are required for the unambiguous structural identification of such compounds and the verification of their role in sexual incompatibility-induced pollen tube arrest. It is, however, difficult not to overestimate the labour and plant biomass requirements that can provide sufficient and adequately pure analyte from such a small plant organ from a non-typical model organism (fruit tree) for the ultimate structural identification. An indirect approach, that is, systematically knocking out genes and checking for the disappearance of a metabolite can only be addressed in model plants with short life cycles (Blomstedt et al. 2016). On the other hand, our study revealed for the first time with an LC–MS based metabolomic approach that such putative metabolites may significantly contribute to the sexual compatibility reactions.

## Electronic supplementary material

Below is the link to the electronic supplementary material.

Electronic supplementary material 1 (PPTX 126 kb)
