# Supplementary material for: LC–MS based metabolic fingerprinting of apricot pistils after self-compatible and self-incompatible pollinations
Source: Plant Mol Biol. 2020 Dec 9;105(4):435–47. doi: 10.1007/s11103-020-01098-5 (PMC7892686; doi:10.1007/s11103-020-01098-5)
Supplement: Supplementary file 1 — Electronic supplementary material 1 (PPTX 126 kb) [file 11103_2020_1098_MOESM1_ESM.pptx]

## Slide 1
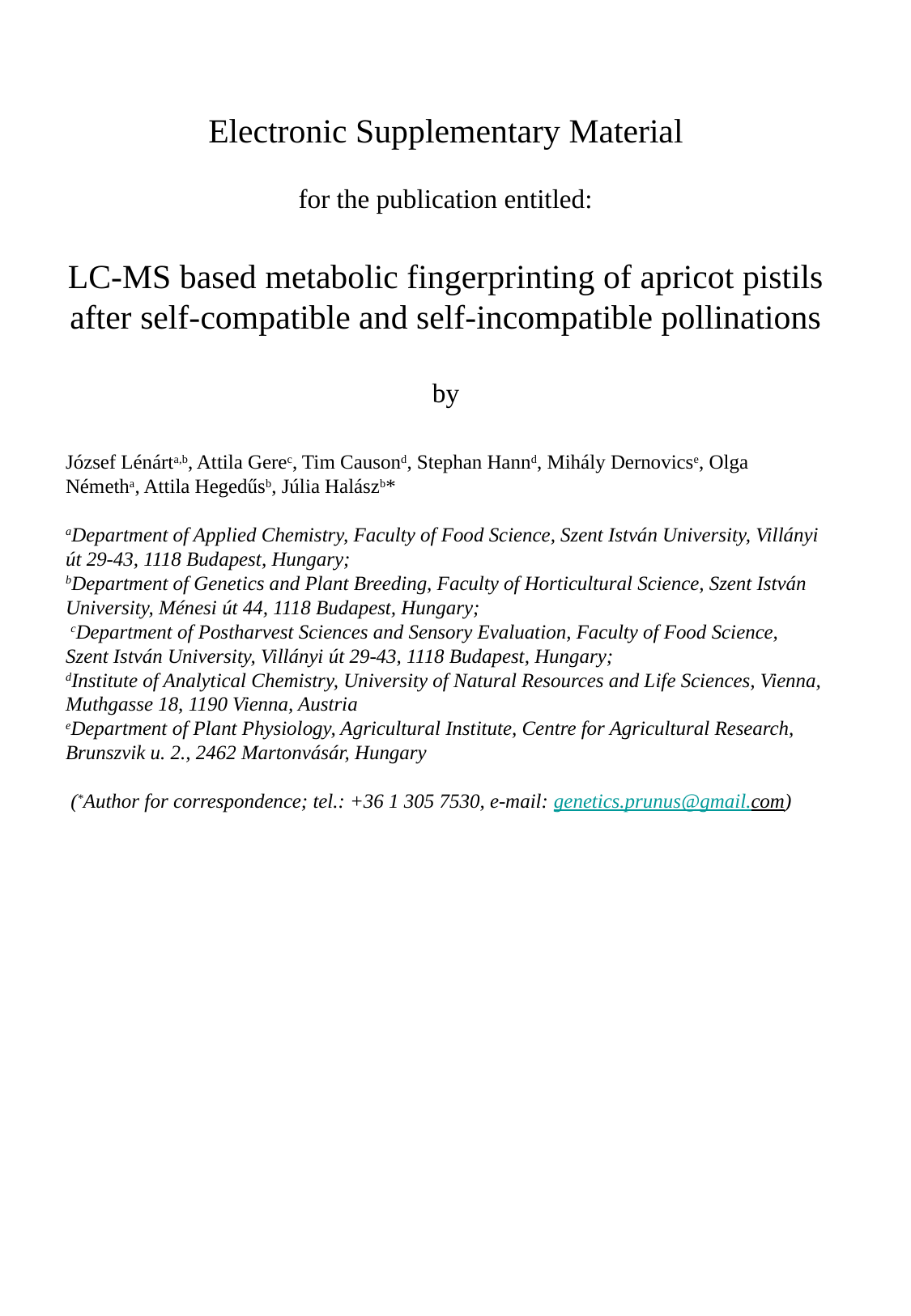

Electronic Supplementary Material
for the publication entitled:
LC-MS based metabolic fingerprinting of apricot pistils after self-compatible and self-incompatible pollinations
 by
József Lénárta,b, Attila Gerec, Tim Causond, Stephan Hannd, Mihály Dernovicse, Olga Németha, Attila Hegedűsb, Júlia Halászb*
aDepartment of Applied Chemistry, Faculty of Food Science, Szent István University, Villányi út 29-43, 1118 Budapest, Hungary;
bDepartment of Genetics and Plant Breeding, Faculty of Horticultural Science, Szent István University, Ménesi út 44, 1118 Budapest, Hungary;
 cDepartment of Postharvest Sciences and Sensory Evaluation, Faculty of Food Science, Szent István University, Villányi út 29-43, 1118 Budapest, Hungary;
dInstitute of Analytical Chemistry, University of Natural Resources and Life Sciences, Vienna, Muthgasse 18, 1190 Vienna, Austria
eDepartment of Plant Physiology, Agricultural Institute, Centre for Agricultural Research, Brunszvik u. 2., 2462 Martonvásár, Hungary
 (*Author for correspondence; tel.: +36 1 305 7530, e-mail: genetics.prunus@gmail.com)

## Slide 2
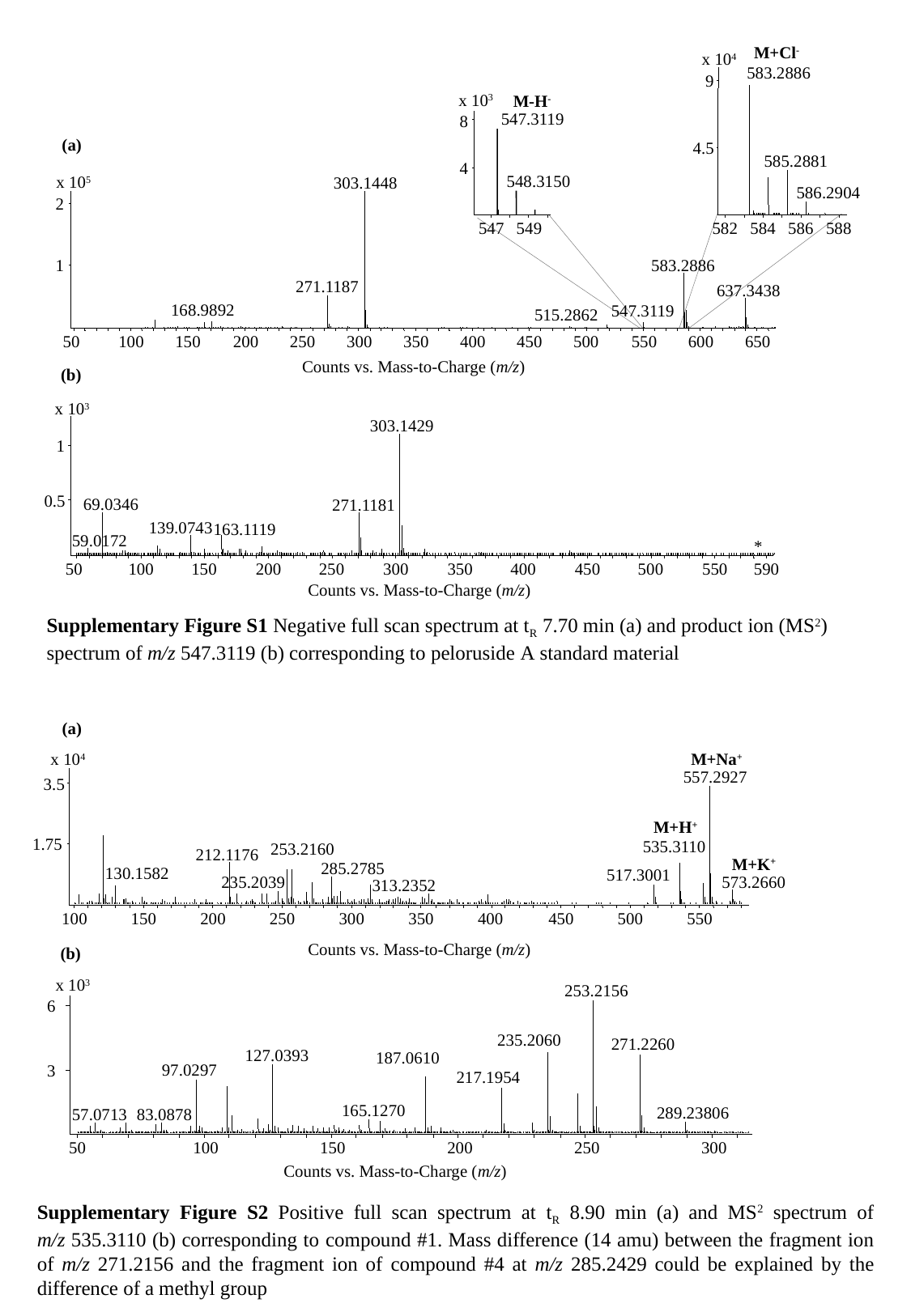

M+Cl-
x 104
583.2886
9
M-H-
x 103
547.3119
8
(a)
4.5
585.2881
4
548.3150
x 105
303.1448
586.2904
2
582
584
586
588
547
549
583.2886
1
271.1187
637.3438
168.9892
547.3119
515.2862
50
100
150
200
250
300
350
400
450
500
550
600
650
Counts vs. Mass-to-Charge (m/z)
(b)
x 103
303.1429
1
0.5
69.0346
271.1181
139.0743
163.1119
59.0172
*
50
100
150
200
250
300
350
400
450
500
550
590
Counts vs. Mass-to-Charge (m/z)
Supplementary Figure S1 Negative full scan spectrum at tR 7.70 min (a) and product ion (MS2) spectrum of m/z 547.3119 (b) corresponding to peloruside A standard material
(a)
x 104
M+Na+
557.2927
3.5
M+H+
1.75
535.3110
253.2160
212.1176
M+K+
285.2785
130.1582
517.3001
573.2660
235.2039
313.2352
100
150
200
250
300
350
400
450
500
550
(b)
Counts vs. Mass-to-Charge (m/z)
x 103
253.2156
6
235.2060
271.2260
127.0393
187.0610
97.0297
3
217.1954
165.1270
289.23806
57.0713
83.0878
50
100
150
200
250
300
Counts vs. Mass-to-Charge (m/z)
Supplementary Figure S2 Positive full scan spectrum at tR 8.90 min (a) and MS2 spectrum of
m/z 535.3110 (b) corresponding to compound #1. Mass difference (14 amu) between the fragment ion of m/z 271.2156 and the fragment ion of compound #4 at m/z 285.2429 could be explained by the difference of a methyl group

## Slide 3
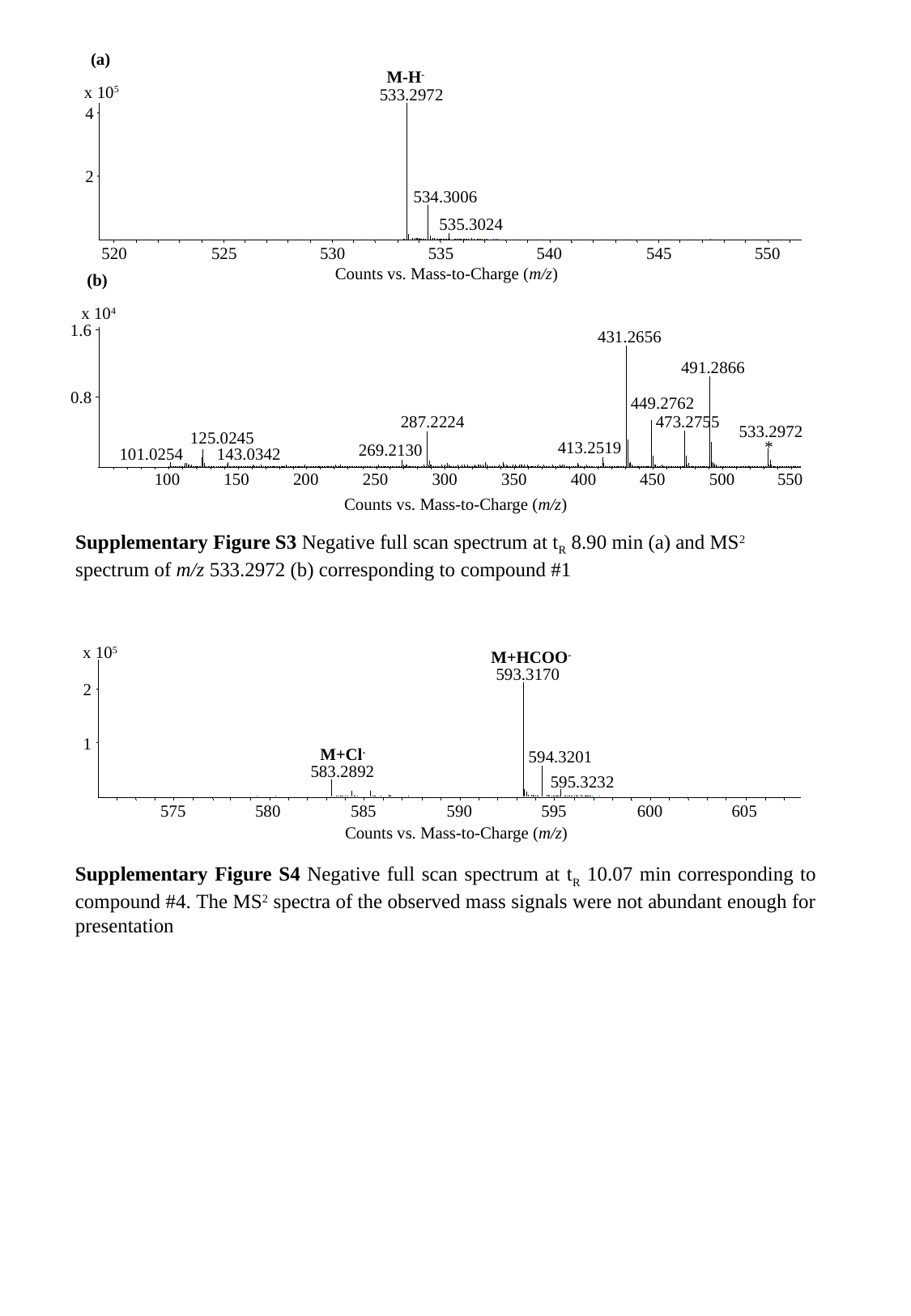

(a)
M-H-
x 105
533.2972
4
2
534.3006
535.3024
520
525
530
535
540
545
550
Counts vs. Mass-to-Charge (m/z)
(b)
x 104
1.6
431.2656
491.2866
0.8
449.2762
287.2224
473.2755
533.2972
125.0245
*
413.2519
269.2130
101.0254
143.0342
100
150
200
250
300
350
400
450
500
550
Counts vs. Mass-to-Charge (m/z)
Supplementary Figure S3 Negative full scan spectrum at tR 8.90 min (a) and MS2 spectrum of m/z 533.2972 (b) corresponding to compound #1
x 105
M+HCOO-
593.3170
2
1
M+Cl-
594.3201
583.2892
595.3232
575
580
585
590
595
600
605
Counts vs. Mass-to-Charge (m/z)
Supplementary Figure S4 Negative full scan spectrum at tR 10.07 min corresponding to compound #4. The MS2 spectra of the observed mass signals were not abundant enough for presentation

## Slide 4
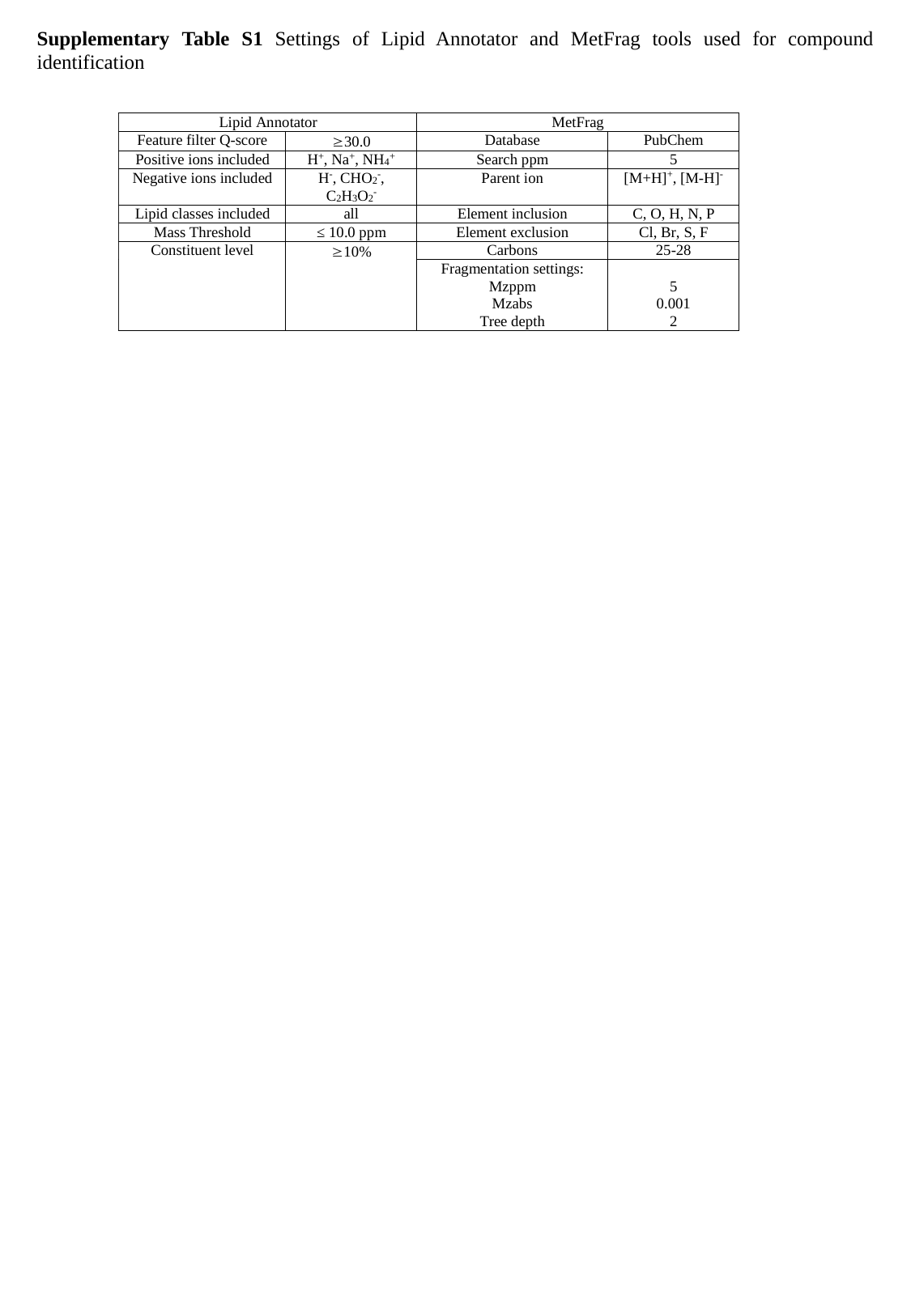

Supplementary Table S1 Settings of Lipid Annotator and MetFrag tools used for compound identification

## Slide 5
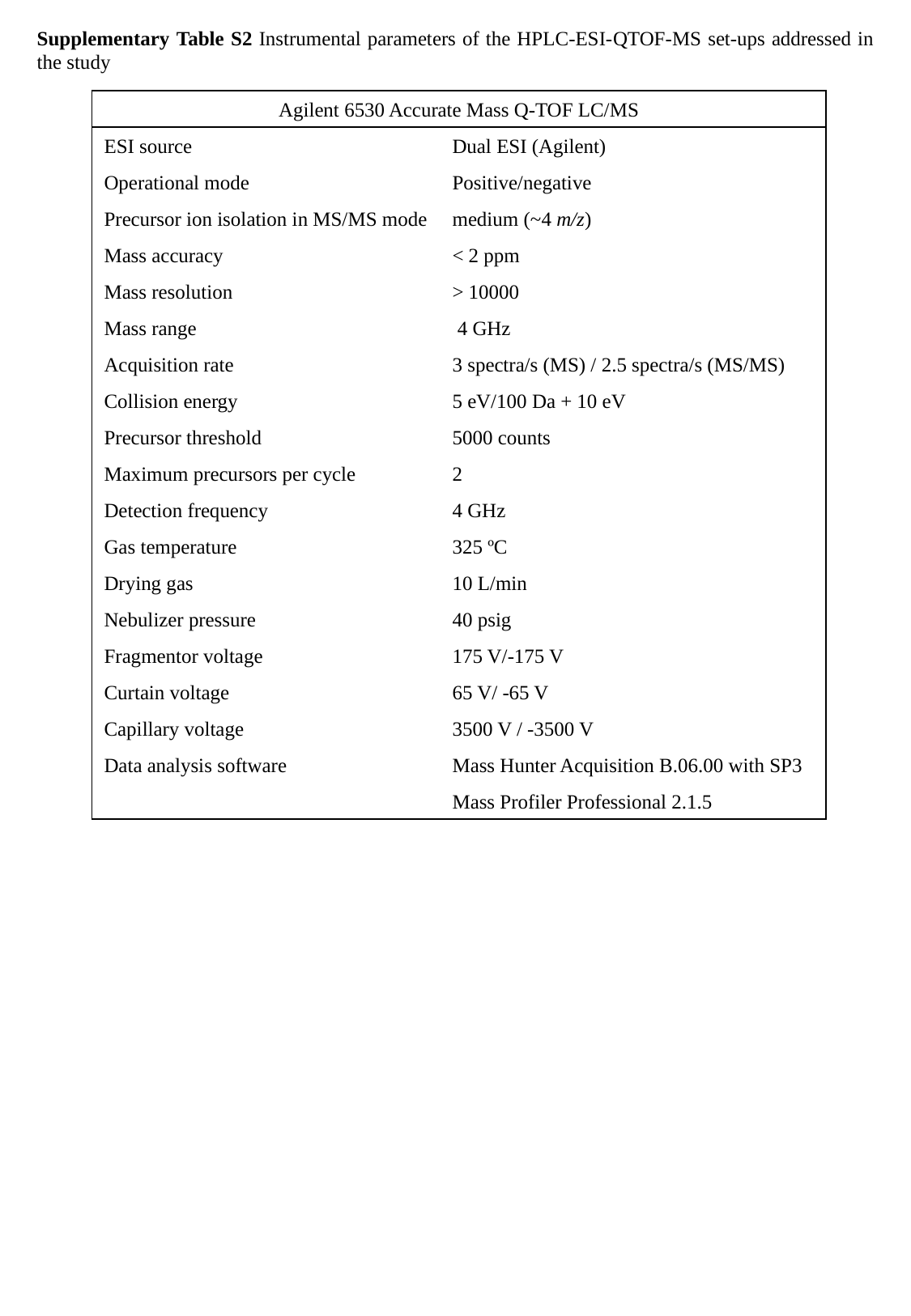

Supplementary Table S2 Instrumental parameters of the HPLC-ESI-QTOF-MS set-ups addressed in the study
| Agilent 6530 Accurate Mass Q-TOF LC/MS | |
| --- | --- |
| ESI source | Dual ESI (Agilent) |
| Operational mode | Positive/negative |
| Precursor ion isolation in MS/MS mode | medium (~4 m/z) |
| Mass accuracy | < 2 ppm |
| Mass resolution | > 10000 |
| Mass range | 4 GHz |
| Acquisition rate | 3 spectra/s (MS) / 2.5 spectra/s (MS/MS) |
| Collision energy | 5 eV/100 Da + 10 eV |
| Precursor threshold | 5000 counts |
| Maximum precursors per cycle | 2 |
| Detection frequency | 4 GHz |
| Gas temperature | 325 ºC |
| Drying gas | 10 L/min |
| Nebulizer pressure | 40 psig |
| Fragmentor voltage | 175 V/-175 V |
| Curtain voltage | 65 V/ -65 V |
| Capillary voltage | 3500 V / -3500 V |
| Data analysis software | Mass Hunter Acquisition B.06.00 with SP3 |
| | Mass Profiler Professional 2.1.5 |
